# Smoking and the Screen Celebrities

**DOI:** 10.4103/0970-2113.44120

**Published:** 2008

**Authors:** Surinder K. Jindal

**Affiliations:** Editor, Lung India (From “Quit Smoking : Why and How?” Publ. Vitasta Publishing Pvt. Ltd., New Delhi, 2008) www.vitastapublishing.com

It is a strange paradox that screen celebrities, who were traditionally considered as responsible for popularizing smoking especially amongst the teens, have themselves suffered the most from the hazards of tobacco. The number of famous actors, directors, singers, producers and other cine-artists who have prematurely died or immensely suffered from tobacco related diseases are almost endless. The list of known celebrity-victims of tobacco is perhaps the biggest of all other similar lists. This was also responsible for one of the toughest ‘nail in the coffin’ for the tobacco trade -- at least in the United States.

Hollywood actors and actresses Lucille Ball, Arnoz Desi, Humphrey Bogart, Richard Boone, Yul Brynner, Rose Cipollone, Gary Cooper, and many others had succumbed to lung or throat cancer, mostly in their 5th to 7th decades of life. Walt Disney, the famous creator of animated characters and producer of Disney films had died of acute circulatory collapse following surgery to remove his lung cancer. Jean Vander Pyl who acted in the famous cartoon series Flintstones, was also a similar victim. His son, Michael O'Meara had later said:

“Everybody on the *Flintstones* smoked and all of them ended up dying of smoking related diseases….. That little cute laugh that Berry and Wilma did with their mouths closed ? They came up with that because when they laughed normally, being smokers, they coughed.”

Jim Varney, the famous actor who died of lung cancer at age fifty was hopelessly hooked on cigarettes. But “he wouldn't allow himself to be photographed smoking for the sake of all the kids who loved Ernest.” And, though he entertained them by clowning, sprawling, grinning and cutting up, the talented Mr. Varney had one last message for those kids: ‘Don't smoke’. Back home in India, the ‘clown story’ is similar to that of the brilliant actor-producer Raj Kapoor of *‘Mera Nam Joker’* fame, who had developed emphysema of the lungs and died of respiratory failure.

Numerous other people in the film industry had suffered from the illnesses caused by tobacco smoking. The long list includes many singers, musicians, designers, choreographers, writers, directors and producers. There are also historians, authors, philosophers, executives, political leaders and kings who have lost their battles against smoking. It might surprise us now that winners like Ayn Rand, the author of such classics as the *‘The fountain-head’ and ‘Atlas Shrugged’*, philosophers like Jean-Paul Sartre and George Harrison, the *‘Quiet Beatle’*, had all failed on the smoking front. Accepting defeat from this formidable enemy, they had fallen at the end but not without a lesson for their fans and their followers. ‘Never again’ was the clear message by all of them. Victor Crawford, who was a tobacco lobbyist and had coined the phrase ‘Health Nazis’ for those against smoking, had turned to advocate tobacco control after he was diagnosed to suffer from lung cancer.

One of the most shocking story is told in the Hollywood movie, *‘The Insider’* which deals with the true story of Dr. Jeffrey Wigand, a former Vice-president of Research and Development in the tobacco industry. It showed that the industry had long known about the health effects of cigarettes. The industry tried to maximize addiction and expand its markets regardless of the truth about the health effects. Wigand was fired and terrorized by the industry for his exposure of the truth.

The consequences of smoking have transformed the lives of several celebrities who have subsequently taken up the cause of anti-tobacco activism, as reformed smokers. Amanda Blake, *‘the Miss Kitty on Gun smoke’*, had developed cancer of her tongue at 48 years of age. She was operated upon, re-learned to speak and widely toured for the American Cancer Society spreading the message of no-smoking very loud and clear. She fought with cancer for about 12 years and died at 60 years of age. President Reagan presented her with the American Cancer Society's ‘Courage Award’ in 1984. Stories of courage and conviction like that of Amanda Blake serve as fountain heads to spread the message of fighting with courage and living a healthy lifestyle.

Possibly, the armamentarium against smoking and tobacco was not as strong in the past as it is now. Many of the earlier falls of our heroes and role-models to smoking could be attributed to ignorance about the harms and lack of adequate methods to handle the problem. Also, the advocates of a healthy life were either too few or too weak. It is no more so. Tobacco is already on the run now.

